# Evaluation of antibacterial efficacy of garlic (*Allium sativum*) and ginger (*Zingiber officinale*) crude extract against multidrug-resistant (MDR) poultry pathogen

**DOI:** 10.5455/javar.2023.j664

**Published:** 2023-06-30

**Authors:** Zakaria Al Noman, Tasnia Tabassum Anika, Sabbya Sachi, Jannatul Ferdous, Yousuf Ali Sarker, Md. Abdus Sabur, Md. Tanvir Rahman, Mahmudul Hasan Sikder

**Affiliations:** 1 Department of Pharmacology, Faculty of Veterinary Science, Bangladesh Agricultural University, Mymensingh, Bangladesh; 2 Department of Microbiology and Hygiene, Faculty of Veterinary Science, Bangladesh Agricultural University, Mymensingh, Bangladesh

**Keywords:** Multidrug-resistant pathogen, alternative antibiotic, TLC-Bioautography

## Abstract

**Objective:**

The study is aimed to understand the antibacterial sensitivity of native and Indian varieties of garlic (*Allium sativum*) and ginger (*Zingiber officinale*) crude extracts against multidrug-resistant (MDR) poultry pathogen (*Escherichia coli *and* Salmonella *sp.).

**Materials and Methods:**

Thin layer chromatography (TLC) is used to identify the target spices’ bioactive antibacterial compounds. MDR *E. coli *and* Salmonella *sp. were isolated from poultry. The TLC-Bioautography technique was applied to explore the antibacterial potentiality of garlic and ginger.

**Results:**

Inhibitory activities of garlic were Zone of inhibition (ZI) = 14.03 ± 0.15 mm and 19.70 ± 0.36 mm, Minimum inhibitory concentration (MIC): 0.625 and 0.325 mg/ml, and ginger were ZI = 14.63 ± 0.30 mm and 11.56 ± 0.51mm, MIC: 9.0 mg/ml against *E. coli and Salmonella *sp., respectively. Two bands of garlic (*R*_f_ value = 0.31 and 0.50) and one band of ginger (*R*_f_ value = 0.71) showed inhibitory potential in TLC-Bioautography against both MDR isolates.

**Conclusion:**

Garlic and ginger were effective against MDR *E. coli *and *Salmonella *sp*. *These spices could be a suitable alternative during the antibiotic void.

## Introduction

Antimicrobial resistance (AMR) is a natural occurrence [1], and numerous factors contribute to the emergence and dissemination of AMR. These factors encompass the indiscriminate utilization of antimicrobial drugs, substandard drug quality [2], and their inappropriate application in agriculture or livestock as feed additives and growth promoters [3].

Globally, 700,000 people die each year from complications associated with AMR, and more than $100 trillion in lost output is anticipated by 2050 [4]. In Asia, uneven antibiotic use and poor public health standards promote AMR, ultimately leading to multidrug resistance (MDR) [5]. Globally, MDR is emerging as a crisis. Bangladesh is also highly susceptible to MDR because of the extensive use of antibiotics and the easy accessibility of over-the-counter antibiotics for both humans and animals [6–8].

The increasing threat of AMR and the decreasing effectiveness of traditional antibacterial agents have led to the need for new and promising antimicrobials. However, there has been a lack of accessible options in development over the past 30 years [9]. It is now crucial to explore alternative approaches, such as vaccines, phage therapy, probiotics, prebiotics, immune modulators, trace elements, and bioactive phytochemicals, to rejuvenate the development of effective treatments against multidrug-resistant (MDR) pathogens [10]. These non-conventional alternatives can help fill the gap left by declining antibiotic effectiveness [11].

Phytochemicals are highly regarded as excellent alternatives due to their cost-effectiveness, ready availability, ease of use, low toxicity, rapid degradability, and environmentally friendly nature [12]. The antibacterial properties of various plants have been documented, which can vary depending on factors such as plant type, composition, quantity utilized, specific microorganism targeted, pH level, and environmental temperature [13].

Garlic (*Allium sativum*) and ginger (*Zingiber officinale*) are recognized as safe and promising alternatives to conventional treatments for various ailments, including diabetes, hypertension, cardiac, neurological, inflammatory, renal, and dental disorders, as well as certain types of cancer [14]. Studies conducted elsewhere have reported on the antimicrobial properties of garlic and ginger [15,16]. These two spices hold an integral role in traditional Asian cuisine, benefiting from favorable geo-climatic conditions that support their cultivation. Medicinally significant plants and spices are frequently employed in healthcare and veterinary practices throughout Asia [17]. While there is evidence supporting the antibacterial efficacy of garlic and ginger, there is a lack of sufficient evidence regarding their effectiveness against MDR pathogens in poultry. Therefore, our study aimed to assess the antibacterial potential of garlic and ginger against MDR bacteria isolated from poultry, as well as to explore their suitability as antibiotic alternatives against MDR bacteria.

## Materials and Methods

### Sample collection

The garlic and ginger utilized in the experiment, including Indian and local varieties, were procured from the nearby Kamal Ranjit (K.R) market. These spices were certified by the Department of Crop Botany, Faculty of Agriculture, Bangladesh Agricultural University.

### Bacterial strains

The strains of *E. coli* and *Salmonella *spp*.* isolated from poultry were acquired from the Department of Microbiology and Hygiene, BAU. Both strains were revived and verified from the stock solution as *E. coli *and *Salmonella *sp*.* on eosin methylene blue agar and Salmonella-Shigella medium, respectively.

### Preparation of extracts

The fresh garlic and ginger were washed properly by running tap water and peeled and sliced into small pieces and ground with mortar and pastel for crude extract, and filtered the extract through Whatman 0.1 mm filter paper. The filtered were collected in a falcon tube, labeled, and stored at 4°C until use.

### MAR index

The MAR (Multiple Antibiotic Resistance) index is determined by dividing the number of resistant antibiotics by the total number of antibiotics tested (AT) against the bacterial isolates under investigation [18], as illustrated in Table 1. An index equal to or greater than 0.2 is indicative of a “high-risk” source of contamination or highly antibiotic-resistant isolates, commonly referred to as superbugs [19].

### Culture preparation and antibiogram

The isolates were subjected to an antibiogram using the disk diffusion test on Mueller Hilton media (Hi-Media, India), following the guidelines provided by the Clinical and Laboratory Standards Institute (CLSI) [20], to confirm their classification as MDR, as shown in Table 2. The disk diffusion test assessed the antibacterial activity of garlic and ginger against MDR *E. coli* and *Salmonella *spp*.* For this, disks with a diameter of 6 mm were prepared using filter paper (Whatman 0.1 mm) and impregnated with the spice extracts. These disks were then placed on Mueller Hinton agar plates inoculated with the bacteria [21]. The diameter of the resulting zones of inhibition was measured in ml.

### Determination of minimum inhibitory concentration (MIC)

The MIC was determined following the method with slight modifications [22]. Dilutions of ginger and garlic extracts were prepared using a two-fold dilution series. These dilutions were then plated on Mueller Hinton agar plates containing MDR *E. coli* or *Salmonella *spp*.* at a standardized concentration of 0.5 McFarland. The plates were incubated at 37°C for 12 h, and the resulting zones of inhibition were observed. The concentration that exhibited the smallest inhibition zone was recorded as the MIC.

**Table 1:** MAR index value of *Escherichia coli *and *Salmonella* spp.

Isolates	MAR value	Resistant Abs	Classification of antibiotics tested														
			Access							Watch							Reserve
			AMP	TE	K	N	O	C	GN	AZM	CIP	E	ETP	S	IMP	MEM	CL
*E. coli*	0.53	8	R	R	S	S	R	R	S	R	S	R	S	R	S	S	R
*Salmonella*	0.46	7	R	R	S	S	R	R	S	R	S	R	S	R	S	S	S

R = Resistant, S = Sensitive, Ampicillin (AMP-2 μg), Azythromycin (AZM-15 μg), Chloramphenicol (C-10 μg), Ciprofloxacin (CIP-5 μg), Colistin (CL-10 μg), Erythromycin (E-15 μg), Etrapenem (ETP-10 μg), Gentamycin (GN-10 μg), Imipenem (IPM-10 μg ), Kanamycin (K-30 μg), Meropenem (MEM-10 μg ), Neomycin (N-30 μg), Oxytetracycline (O-30 μg), Streptomycin (S-5 μg), Tetracycline (TE-30 μg).

**Table 2.** Antibacterial ZI and MIC of garlic and ginger.

Bacterial isolates	ZI (mm)				MIC (mg/ml)	
	Garlic		Ginger		Garlic	Ginger
	Indian	Local	Indian	Local	Indian	Indian
*Escherichia coli*	14.03 ± 0.15	0	14.63 ± 0.30	0	0.625	9.0
*Salmonella *spp*.*	19.70 ± 0.36	0	11.56 ± 0.51	0	0.312	9.0

### Thin layer chromatography(TLC)-Bioautography

A 10 μl portion of the crude extract from garlic and ginger was applied to a silica-coated thin TLC plate (Merck, silica gel 60 F254) in duplicate. The TLC plates were developed using a mobile phase composed of a mixture of glacial acetic acid, propanol, water, and ethanol in equal proportions (20:20:20:20) and were visualized under a UV light at 254 nm. The *R*_f_ value (retention factor) of the chromatograms was determined for comparison purposes. The *R*_f_ value represents the ratio of the distance traveled by the compound from its origin to the movement of the solvent from the origin. The bands on the TLC plate were then carefully separated using scissors [23]. Each band from garlic and ginger extracts was inoculated into cultures containing bacteria and incubated at 37°C for 24 h to observe the resulting outcome [24].

### Statistical analysis

The statistical software IBM Statistical Package for Social Sciences Statistics 20.0 was utilized to analyze the mean ± SD values obtained from three replicates. Each experiment was repeated three times, and consistent results were obtained in each instance.

## Results

### MAR index value

The MAR values for MDR *E. coli* and *Salmonella *spp*.* are 0.53 (against eight antibiotics) and 0.46 (against seven antibiotics), respectively, as shown in Table 1. Among the 15 AT for resistance, both bacterial isolates exhibited resistance to seven antibiotics, except MDR *Salmonella *spp*.*, which showed susceptibility to colistin. The results are categorized according to the WHO AWaRe categorization [25]. Among the seven antibiotics in the Access group, both isolates demonstrated resistance to four antibiotics (4/7, 57% resistance). For the seven antibiotics in the Watch group, both isolates showed resistance to three antibiotics (3/7, 42%). In the Reserve group, representative antibiotic colistin was tested, and only *E. coli* exhibited resistance.

**Table 3.** TLC-Bioautography of garlic and ginger.

Bacterial isolates	*R*_f_ value	Band color	Antibacterial potency
Standard	A= 0.68	Blue	A
Garlic	A= 0.3 B= 0.31 C= 0.50 D= 0.69	Blue	B and C ( B > C)
Ginger	A= 0.69 B= 0.71	Greenish yellow	B

Standard, Ciprofloxacin = 100 μg/ml.

**Figure 1.**
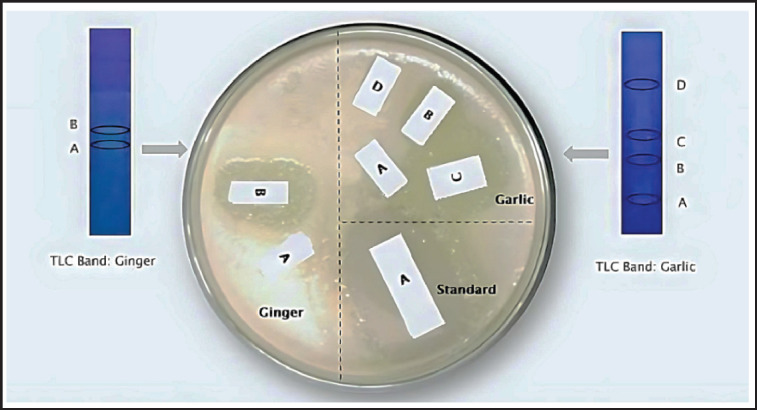
Phytochemical analysis by TLC-bioautography of crude extract of ginger (left panel) and garlic (right panel). Two bands (A & B) and four bands (A, B, C, and D) were separated. Only B band of ginger and B & C bands of garlic shows antibacterial activity.

### Antibacterial efficacy of garlic and ginger

The antibacterial efficacy of garlic and ginger against MDR *E. coli* and *Salmonella *spp*.* are arranged as a zone of inhibition (ZI) and MIC, presented in Table 2. Garlic and ginger of the Indian variety show intermediate antibacterial efficacy according to CLSI standard [20], though no efficacy was shown by the local variety in both ginger and garlic. Garlic extract shows more efficacy towards MDR *Salmonella *spp. than *E. coli*, whereas ginger shows similar efficacy in both MDR strains.

### Phytochemical analysis in (TLC)-Bioautography

During the TLC analysis, four distinct bands (A, B, C, and D) were observed in the garlic extract, while two separate bands (A and B) were identified in the ginger extract, as indicated in Table 3. The garlic extract displayed blue bands, whereas the ginger extract exhibited greenish-yellow bands. Among the bands, B and C of the garlic extract and the B band of the ginger extract demonstrated antibacterial activity, as illustrated in Figure 1.

## Discussion

Our objective was to assess the effectiveness of native and Indian varieties of garlic and ginger against MDR bacteria isolated from poultry and to explore their potential as alternative treatments to combat MDR bacteria. We examined the MAR values of both bacterial strains. The MAR values of the *E. coli* and Salmonella sp. isolates were significantly higher than the threshold of 0.2, indicating that both species possess high-risk characteristics with superbug capabilities, posing significant risks to human and veterinary health. Additionally, we observed that the isolated MDR poultry pathogens exhibited resistance to antibiotics in the Access, Watch, and Reserve groups, as per the WHO AWaRe categorization [25]. However, it is important to note that various factors contribute to developing MDR, including the indiscriminate use of antibiotics, environmental stress, genetic factors, and more [21].

In this study, we focused on evaluating the antibacterial efficacy of the crude aqueous extract. The use of aqueous extract has been traditionally practiced by local healers and is commonly employed by inhabitants of the Indian subcontinent [26]. The disk diffusion test was conducted to assess the antibacterial effectiveness, and both Indian garlic and ginger exhibited intermediate efficiency of antibiotics based on the CLSI [20] standard, which aligns with previous research findings [27–29]. However, it is important to note that the disk diffusion method may not always accurately demonstrate the potency of plant extracts, especially those that are relatively non-polar. The aqueous agar matrix utilized in agar diffusion experiments does not disperse well with these compounds [30]. In our study, the local varieties of spices did not demonstrate potency. This could be attributed to factors such as differences in bioactive compound concentrations, as local varieties may differ from the tested variety [31]. Additionally, variations in climatic conditions, soil conditions, environmental factors, and agricultural techniques could contribute to the differences in potency [32,33].

The antimicrobial properties of garlic primarily rely on the presence of allicin, a thiosulfate compound found in crushed garlic bulbs [34], as well as antibacterial sulfur bio-compounds like alliin and alliinase [35,36]. On the other hand, ginger’s antimicrobial activity can be attributed to its essential oil or oleoresins [37], as well as phenolic compounds such as eugenol, shogaols, zingerone, gingerdiols, gingerols, and their synergistic interactions with other compounds like β-sesquiphellandrene, cis-caryophyllene, zingiberene, α-farnesene, α- and β-bisabolene [36,38]. However, it is important to note that the antimicrobial activity of garlic and ginger can vary depending on factors such as chemical composition, extraction solvent, methodology, and processing techniques [37].

The effectiveness of a plant extract against a particular pathogen is influenced by both plant-related factors and pathogen-related factors. Plant-related factors encompass genetic variations, spice quality, environmental conditions, species, breeds, varieties, processing techniques, pH of the extract, moisture content, and concentration of active ingredients, as well as climatic and environmental factors such as temperature, soil quality, water levels, sunlight, wind direction, and other factors associated with production and storage [39,40]. Pathogen-related factors include genetic variations within the isolate and strain, variations in drug resistance, the host’s characteristics, infection load, and severity, among others, which can influence antibacterial efficacy. In our study, since we tested the same strains, pathogen-related factors were not a variable; however, when comparing our study with others, pathogen-related factors could be variable, leading to differences in the MAR value. In diseased conditions, the host’s factors also play a significant role in determining effective outcomes, including disease status, immunity level, physiological condition, genetic factors, and lifestyle [40].

Using the TLC-Bioautography technique for phytochemical analysis offers a rapid, straightforward, and cost-effective approach to assess the antibacterial properties of separated bands or fractions derived from natural products. This technique combines chromatographic separation with a bio-assay, enabling the detection, identification, and isolation of bioactive constituents from plants or spices [41]. In our study, the TLC technique was employed to screen various bioactive compounds in garlic and ginger, yielding results consistent with previous research findings [23]. However, we did not specifically identify the active ingredients responsible for the observed antibacterial activity. The phytochemical analysis of different bands from both garlic and ginger revealed varying levels of antibacterial efficacy. Overall, our study demonstrates the potential of garlic and ginger as promising chemotherapeutic agents for combating diseases caused by MDR pathogens.

## Conclusion

MDR strains of *E. coli* and *Salmonella *spp*.* pose significant concerns for both human and animal health. This study proves that the Indian variety of garlic and ginger, but not the local variety, exhibits antibacterial efficacy against MDR *E. coli* and *Salmonella *spp*.* strains isolated from poultry. However, further investigation is required to identify the specific bioactive constituents responsible for their antibacterial properties. Additionally, determining the effective dosage, potential side effects, and toxicity profile of garlic and ginger is crucial for understanding their pharmacokinetics and pharmacodynamics.

## List of Abbreviations

AMR, Antimicrobial resistance; CLSI, Clinical and Laboratory Standards Institute; MDR, Multi drug-resistant; MIC, Minimum inhibitory concentration; Rf value, Retention factor value; TLC, Thin Layer Chromatography; ZI, Zone of inhibition
